# Characterization of Two Ethephon-Induced *IDA-Like* Genes from Mango, and Elucidation of Their Involvement in Regulating Organ Abscission

**DOI:** 10.3390/genes12030439

**Published:** 2021-03-19

**Authors:** Avinash Chandra Rai, Eyal Halon, Hanita Zemach, Tali Zviran, Isaac Sisai, Sonia Philosoph-Hadas, Shimon Meir, Yuval Cohen, Vered Irihimovitch

**Affiliations:** 1 The Volcani Center, Institute of Plant Sciences, Agricultural Research Organization (ARO), Rishon LeZion 7528809, Israel; avinash@volcani.agri.gov.il (A.C.R.); eyalh@volcani.agri.gov.il (E.H.); hanita@volcani.agri.gov.il (H.Z.); taliz@volcani.agri.gov.il (T.Z.); sissai@volcani.agri.gov.il (I.S.); vhyuvalc@volcani.agri.gov.il (Y.C.); 2 The Volcani Center, Department of Postharvest Science, Agricultural Research Organization (ARO), Rishon LeZion 7528809, Israel; vtsoniap@volcani.agri.gov.il (S.P.-H.); shimonm@volcani.agri.gov.il (S.M.)

**Keywords:** abscission zone, cell wall genes, cytosolic pH, ethephon, ethylene signaling, fruitlet-bearing explants/trees, IDA/IDL, mango (*Mangifera indica* L.)

## Abstract

In mango (*Mangifera indica* L.), fruitlet abscission limits productivity. The INFLORESCENCE DEFICIENT IN ABSCISSION (IDA) peptide acts as a key component controlling abscission events in Arabidopsis. IDA-like peptides may assume similar roles in fruit trees. In this study, we isolated two mango *IDA-like* encoding-genes, *MiIDA1* and *MiIDA2*. We used mango fruitlet-bearing explants and fruitlet-bearing trees, in which fruitlets abscission was induced using ethephon. We monitored the expression profiles of the two *MiIDA-like* genes in control and treated fruitlet abscission zones (AZs). In both systems, qRT-PCR showed that, within 24 h, both *MiIDA-like* genes were induced by ethephon, and that changes in their expression profiles were associated with upregulation of different ethylene signaling-related and cell-wall modifying genes. Furthermore, ectopic expression of both genes in Arabidopsis promoted floral-organ abscission, and was accompanied by an early increase in the cytosolic pH of floral AZ cells—a phenomenon known to be linked with abscission, and by activation of cell separation in vestigial AZs. Finally, overexpression of both genes in an *Atida* mutant restored its abscission ability. Our results suggest roles for *MiIDA1* and *MiIDA2* in affecting mango fruitlet abscission. Based on our results, we propose new possible modes of action for IDA-like proteins in regulating organ abscission.

## 1. Introduction

Abscission is a unique developmental process, which facilitates the detachment of excess, damaged, or no longer needed plant organs [1,2,3]. Abscission of plant organs, including leaves, flowers, and fruits, is achieved by the degradation of the middle lamella localized between differentiated cells in specialized abscission zones (AZ) found at the base of the shedding organs [1,2,3]. Regardless of the variation in the sites of abscission, different abscission processes share similar features at the cellular level. The sequence of events occurring at the AZ is proposed to be divided into four main stages, including: (i) Differentiation of cells in the AZ. (ii) Acquisition of the competence of the AZ cells to respond to the abscission signal/s. (iii) Activation of the process by abscission signals, leading to cell wall loosening and cell separation. (iv) Trans-differentiation of the mother plant-retained AZ, to generate a protective layer [4,5].

Different factors trigger and/or affect abscission events. The two main hormones affecting organ abscission are ethylene, acting as an inducer, and auxin (mainly IAA), acting as a suppressor. While ethylene is suggested to play a central role in initiating abscission events, basipetal IAA flux, through the AZ, is suggested to delay or prevent abscission by reducing the sensitivity of the AZ to ethylene [2,6,7]. Since it was demonstrated that IAA is also required as a prerequisite for organ shedding [8], it is possible that IAA plays distinct roles during the early and later stages of abscission.

Besides plant hormones, small cell-to-cell communicator peptides are also known to act as important signaling molecules that regulate abscission. Especially, genetic studies in Arabidopsis have revealed the importance of the small, secreted peptide INFLORESCENCE DEFICIENT IN ABSCISSION (IDA), and its two leucine-rich repeat receptor-like kinases (LRR-RLKs) targets, HAESA (HAE) and HAESA-like2 (HSL2), in regulating cell separation during floral organ abscission [9,10,11]. Based on these studies, it was proposed that activation of the RLK receptors by the secreted IDA peptide operates through a mitogen-activated protein (MAP) kinase cascade, which in turn activates KNOTTED1-LIKE HOMEOBOX (KNOX) transcription factors, leading to induction of cell wall remodeling and degrading enzymes genes in the AZ [12,13]. Accordingly, it was shown that floral organs of *Atida* mutant plants do not abscise throughout the development of the siliques, while complementation of the *AtIDA* gene under constitutive expression of the 35S promoter restored their floral organ abscission [14]. Moreover, based on the observations that the *Atida* mutants, which display ethylene sensitivity, showed a pronounced delay in the abscission of their floral organs even after exogenous ethylene treatment, it was initially proposed that the IDA-HAE/HSL2 module might operate in an ethylene-independent manner [9,11,14]. This concept, however, has been recently challenged [15,16].

The Arabidopsis *IDA* gene belongs to a small gene family comprising of eight additional *IDA-like* (IDL) members [17]. While five of these genes (*AtIDL1-5*) are able to replace *AtIDA* and promote floral organ abscission to varying degrees [9,18], the *AtIDL6-8* genes have been linked to processes other than plant organ abscission, including stress-related responses [17]. Interestingly, *AtIDA* and *AtIDL1* were also shown to be involved in controlling cell separation processes that occur during lateral root emergence and root cap sloughing [18]. Genes encoding IDA proteins have also been identified in different plant species [19,20]. IDA peptides are evolutionarily conserved across the plant kingdom, presenting especially high similarity in their conserved C-terminal domain (PIP domain), which contains 12 residues constituting the active ligand motif of the secreted peptide [18,21]. Based on differences observed within the PIP domain, eudicot IDA/IDL proteins were initially classified as either AtIDA/IDL1-like, PIP_R_ ([P,L][V,I],PPS[A,G]PSK**R**HN)-type or AtIDL2-5-like, PIP_k_ (PIP[A,T,H,P]S[A,G]PSR**K**HN)-type, based on the residue at position 10 of the PIP domain (arginine or lysine). An additional version of the IDL protein, closely resembling the PIP_k_-types, yet containing glutamine at position 10 (termed PIP_Q_-type), was identified in monocots [19,20]. Lastly, phylogenetic studies also identified HAE/HSL2 orthologues in different flowering plants, suggesting the possible conservation of the IDA-HAE/HSL2 module in controlling cell separation processes in all angiosperms [19,20].

To date, distinct IDL family members, including those from tomato and soybean [22], citrus [23], lychee [24], oil palm [25], yellow lupine [26], and most recently *Nicotiana benthamiana* [27], have been characterized. In terms of their mode of action during plant organ shedding, expression analyses have shown that these genes are highly expressed in leaf, flower, and/or fruit AZs during organ abscission. Notably, the expression of a pair of *NbIDL* homologous genes in leaves and roots was also implicated in response to water stress [27]. It was also demonstrated that overexpression of *IDL*-genes from citrus (*CtIDA3*) and from lychee (*LcIDL1*) in Arabidopsis enhanced its floral organ detachment, while their expression in *Atida2* mutants restored their plant abscission ability [23,24].

Mango (*M. indica*) belongs to the Anacardiaceae family that grow in tropical and sub-tropical climatic zones [28]. Although the global demand for mango is increasing rapidly, production is often constrained by the excess fruitlet and fruit shedding that occurs throughout the growing season [29]. In mango, a pre-differentiated fruitlet AZ exists already from the flowering stage to fruit maturity. It can be detected as a thin circular groove, at the pedicel, close to the fruitlet base [29]. Previous studies showed that natural mango fruitlet drop correlates with high ethylene production in fruitlet tissues [30]. In line with this observation, studies from our and other groups have shown that treatment with the ethylene-releasing compound, ethephon, hastens mango fruitlet abscission [31,32]. Further examination of the expression patterns of two mango ethylene receptor genes, *MiETR1* and *MiERS1*, showed that mango fruitlet shedding is associated with strong upregulation of *MiERS1* in fruitlet AZs [31], and with an increase in the *MiERS1/MiETR1* ratio in the AZs of “about to abscise” fruitlets [32]. These studies were later extended to monitor levels of expression of novel *MiERS1* and *MiETR1* isoforms present in the ‘Hoi’ poly-embryonic mango cultivar [33]. With respect to IAA function, it was shown that mango fruitlet drop is associated with a reduction in the polar IAA transport capacity through the fruitlet pedicel, and with a reduction in sucrose accumulation in the fruitlet pericarp tissue [32]. Lastly, work from our group recently linked reduced expression of specific IAA-carrier genes in fruitlet tissues with a decrease in free IAA content in the AZ [34]. Together, these findings suggest that an increase in ethylene perception in the AZ, in addition to limited sucrose accumulation in fruitlet tissues and reduced IAA supply to the AZ, may induce natural fruitlet drop. Despite the current knowledge, an in-depth exploration of processes related to the control of mango fruitlet drop, is still missing.

In the present study, to explore additional mechanisms regulating mango fruitlet drop, we first isolated two mango *IDA/IDL* encoding genes, *MiIDA1* and *MiIDA2*. We monitored the expression patterns of these genes, together with the expression patterns of different ethylene signaling-related and cell wall modifying genes, in control and ethephon-treated fruitlet AZs taken from two experimental mango systems (explants and trees). Lastly, we examined the ectopic expression of *MiIDA1* and *MiIDA2* in Arabidopsis, and assessed their capacity to restore *Atida* mutant abscission ability. Our results highlight the potential roles of both ethephon-induced *IDA* genes during mango fruitlet abscission, and suggest possible new modes for their action.

## 2. Materials and Methods

### 2.1. Plant Material, Induction of Fruitlet Abscission, and Fruitlet AZ Tissue Sampling

Mature commercially bearing ‘Kent’ mango trees grafted on ‘13-1’ rootstocks were used for the experiments. The trees were grown in the Ramat Magshimim orchard, located in northeast Israel (32°50′ N, 35°48′ E). Tissue sampling of fruitlet AZs was performed separately from fruitlet-bearing explants (FBEs) and from fruitlet-bearing trees (FBTs). For FBE AZ sampling, mango explants (each bearing 1 to 2 fruitlets per panicle) were collected from the orchard early in the morning at the onset of May 2018. The explants were kept in water, and were brought to the laboratory within 2.5 h after collection. In the laboratory, the basal end of each explant was placed in a 50-mL tube containing water, and kept at 25 °C. The explants were then divided into six experimental units, each comprising 100–120 explants. Fruitlet abscission was induced in three experimental units using 1400 µL·L^−1^ ethephon (Ethrel, Agan Chemicals, Ashdod, Israel), containing 0.05% (*v*/*v*) Triton X-100, essentially as described [31,34]. The remaining untreated experimental units served as controls. Fruitlet AZ samples from treated and control units were collected at different time points, frozen in liquid nitrogen, and kept at −80 °C until further analysis. Before sampling, fruitlet counting was performed to calculate abscission rates. For sampling AZs from FBTs, 20 fully heavily loaded trees were selected at the onset of May 2018. The trees were divided into two groups: Ten trees were sprayed with ethephon (1400 µL·L^−1^) using a 2.5-L solution containing 0.05% (*v*/*v*) Triton X-100 per tree, and 10 control trees were sprayed with a solution containing only the surfactant. The concentration of ethephon, (1400 µL·L^−1^), which was used in the field experiment, was chosen based on a preliminary filed trial showing that it was also effective in inducing high abscission rates, in trees in the field. For each treatment, six trees were used for tissue sampling, and four trees were used for abscission rate assessment. Fruitlet AZ sampling was carried out early in the morning. Ten panicles (each bearing at least 1 to 2 fruitlets) were collected from each tree at different time points. Upon dissection, AZ samples were pooled into six groups, each comprising AZs collected from two control or two treated trees. For abscission rate assessments, 15 healthy-appearing panicles were tagged in the remaining treated and untreated trees. The number of fruitlets was counted at different time points, and abscission rates were calculated.

### 2.2. RNA Isolation and cDNA Synthesis

Mango fruitlet AZs were ground in liquid nitrogen using an IKA-A11 analytical grinding mill (IKA-Werke, Staufen, Germany). Total RNA was extracted from 2 g of ground frozen plant material using the hexadecyltrimethyl ammonium bromide (CTAB) method [35]. RNA sample quantities and quality were analyzed using a Bioanalyser 2200 apparatus (Agilent Technologies, Santa Clara, CA, USA). Following confirmation of RNA integrity, 5 µg of total RNA, pre-treated with 1 unit of RQ1 DNase, served as a template in the synthesis of the first-strand cDNA, using an anchored oligo-dT primer and SuperScript III Reverse Transcriptase (Thermo Scientific, Waltham, MA, USA), according to the manufacturer’s instructions. The reaction products were then used for further analyses.

### 2.3. Cloning of MiIDA1 and MiIDA2 Genes

To identify mango sequences encoding proteins similar to the product of *IDA* genes, a nucleotide BLAST search was conducted against a ‘Kent’ mango transcriptome database generated by our group (Rai et al., unpublished), with conserved regions of *AtIDA* and *CiIDA3* RNA serving as query. This search led to identifying two expressed sequence tags (ESTs) putatively encoding IDA proteins. The two EST sequences were next verified by RT-PCR, using specific pairs of end-to-end primers designed within their 5’ and 3’ UTRs, and cDNA synthesized from ‘Kent’ fruitlet AZs (a mixture of samples collected at different intervals) as a template (Supplementary Table S1). The obtained PCR products were ligated into the CloneJET vector (Fermentas, Vilinius, Lithuania), sequenced (Hy-labs Laboratories, Rehovot, Israel), and further used as templates to generate constructs for Arabidopsis transformation purposes (see below).

### 2.4. Selection of Mango Genes for Expression Analysis

For expression studies, distinct ESTs, representing either full-length or partial sequences putatively encoding for different ethylene signaling-related genes, distinct cell wall remodeling and degrading enzymes, and for glyceraldehyde 3-phosphate dehydrogenase (GAPDH), were retrieved from the generated ‘Kent’ mango transcriptome. *MiETR1* (AF227742.1) and *MiERS1* (JF323582.2) sequences were also chosen for analysis. For each selected gene, specific primers were designed using Benchling software (www.benchling.com (accessed on 25 August, 2018)) (Supplementary Table S2). Prior to the expression analyses, the specificity of each pair of primers was validated by PCR using cDNA synthesized from collected AZs (a mixture of samples collected at different time points).

### 2.5. Real-Time Quantitative (q) PCR Analysis

Fruitlet AZ tissues from control and ethephon-treated FBEs or from control and ethephon-treated FBTs were used for RNA extraction. Following RNA extraction and cDNA synthesis, three biological replicates containing two technical replicates for each tissue, treatment, and sampling point, were subjected to analysis. Real-time qPCR was performed at the Biological Services Unit, The Weizmann Institute of Science (Rehovot, Israel) using a BioMark 48.48 Dynamic Array (Fluidigm, San Francisco, CA, USA), following the manufacturer’s ADP37 Fast GE protocol (http://www.fluidigm.com/user-documents (accessed on 10 November 2018)). Briefly, pre-amplification of cDNA products was performed with 1.25 µL samples of 50 ng·µL^−1^ Fluidigm PreAmp Master Mix (Fluidigm, PN 1005581). An aliquot (2.7 µL) of each pre-amplified cDNA was mixed with 3 µL of SsoFast EvaGreen Supermix with Low Rox (BioRad, PN 1725211), and 0.3 µL of 20X Binding Dye Sample Loading Reagent (Fluidigm, PN 1001388). Five µL of each sample mix was pipetted into a sample inlet of a 48.48 Dynamic Array chip. Individual primer pairs (50 µM) in a 1.08 µL volume, mixed with 3 µL Assay Loading Reagent (Fluidigm PN 85000736) and 1.92 µL of Low TE buffer, were pipetted into the second inlet. Subsequent sample/assay loading was performed with the IFC Controller HX (Fluidigm) followed by qPCR performed on the BioMark HD apparatus (Fluidigm). Raw array data were analyzed with Fluidigm Real-Time PCR Analysis software with a manually set threshold of 0.02 and target Ct range of 15 to 35. Relative gene expression (RE) was calculated using *MiGAPDH* as an endogenous reference gene.

### 2.6. Arabidopsis Transformation and Phenotypic Analysis

For constitutive expression of the two *MiIDA* genes in Arabidopsis, the plasmid pART7-based pART27 vector was used. The protein-coding regions of *MiDA1* and *MiIDA2* were first amplified using the *MiIDA1/MiIDA2*-*Xho*I and *MiIDA1*/*MiIDA2*-*Xba*I primers (Supplementary Table S1). The purified PCR fragments were digested with *Xho*I and *Xba*I, respectively, and cloned into the *Xho*I-*Xba*I site of the pART7 vector between the 35S*caMV* promoter and the *ocs* 3’ transcription terminator. The expression cassettes were next *Not*I-excised from the pART7 constructs, and inserted into the binary plant transformation vector, pART27. The resulting plasmids, named pART27 35S:*MiIDA1* and pART27 35S:*MiIDA2,* were further used for stable transformation of wild type (WT) (Col-0) Arabidopsis plants using the *Agrobacterium tumefaciens*-mediated floral dip method [36]. The transformed seeds were selected on medium containing half-strength Murashige and Skoog salts and kanamycin (50 µg/mL). For complementation purposes, an *Atida* T-DNA insertion line, prepared by the Salk Institute Genomic Analysis Laboratory, was obtained from the Nottingham Arabidopsis Stock Centre (NASC). The (SALK_133209.24.65.x) line in a Col-0 background contains a T-DNA insert in the 5′UTR region 6 bp upstream of the translation start codon. Before attempting complementation, the homozygosity of the *Atida* T-DNA insertion plants was confirmed using forward 5’ UTR region, and reverse from 3′ left border repeats primers (Supplementary Table S1). The pART27 35S:*MiIDA1* and pART27 35S:*MiIDA2* constructs were further used for stable transformation of the *Atida* mutant, as described above. Seven and nine independent randomly selected PCR-positive kanamycin-resistant plants obtained (35S:*MiIDAs* and *Atida* 35S:*C_MiIDAs*, respectively), were self-pollinated (F2 progeny), and plants from the second generation of overexpressing, and/or complemented *MiIDAs* lines were further used for phenotypic assessment. All plants were grown in a growth room under a long day regime (25 °C, 16/8 h light/dark). To confirm *MiIDA* expression in the transformed plants, total RNA was isolated from the leaves of *MiIDA*-overexpressing, and/or from *MiIDA*-complemented plants, using a Plant/Fungi Total RNA Isolation Kit (Norgen Biotekcrop, Thorold, ON, Canada). First strand cDNA was generated, and the accumulation of *MiIDA* genes was evaluated by RT-PCR, using Fast SYBR Green Master Mix (Applied Biosystems, Foster City, CA, USA). Reactions were carried out using 3 µL of the cDNA products (1:10 dilution), 6 μL of SYBR Green PCR Master mix, and 200 nM primers from the relevant primer pair in a final volume of 12 µL. Analysis was performed with a One Step One Plus Real Time machine (Applied Biosystems). cDNA samples were analyzed in triplicates. Transcript levels in each sample were estimated using a standard curve for each gene, normalized against the *AtACT* transcript level. Relative expression (RE) levels were calculated by dividing each individual gene copy number by the *AtACT* copy number. For phenotypic characterization, plant heights and siliques lengths (mm) were measured. Flowers and siliques from positions 2 to 9 along inflorescences of WT, *MiIDA*-overexpressing, and *MiIDAs* complemented plants were removed from the plant bodies and imaged using a Binocular (Leica MZFL III) equipped with a Nikon Digital sight camera (DS-Fi1, Japan). All measurements were performed with 3 or 4 biological replicates.

### 2.7. BCECF Fluorescence Analysis

Fluorescence analysis with the fluorescent dye 2′,7′-bis-(2-carboxyethyl)-5(and-6)-carboxy-fluorescein-acetoxymethyl (BCECF-AM) was performed essentially as previously described [37]. Developing flowers and siliques from WT, *MiIDA*-overexpressing, and/or *MiIDAs-*complemented lines, counted from the first flower with visible petals at the top of the inflorescence (positions 2 to 9 along the inflorescences), were examined. All floral parts except carpels, receptacle, and peduncles from flowers or developing siliques, were removed carefully. Samples were immersed in 10 µM BCECF- AM (Thermo Scientific, Inc., Waltham, MA, USA) solution under darkness for 20 min, after which the samples were rinsed 4 to 5 times with phosphate-buffered saline solution (PBS, pH 7.4), to remove excess BCECF. Images were taken with an Olympus Optical IX-81 confocal laser-scanning microscope (CLSM) (FV 500, Olympus Optical, Tokyo, Japan), equipped with a 488 nm argon-ion laser. Samples were excited by 488 nm light, and emission was detected through a BA 505–525 filter. A BA 660 IF emission filter was used to detect chlorophyll auto-fluorescence. Transmitted light images were obtained using Nomarski Differential Interference Contrast (DIC) microscopy. All analyses were performed using 3 or 4 biological replicates.

### 2.8. Scanning Electron Microscopy (SEM)

Floral AZs and vestigial AZs from WT, *MiIDA*-overexpressing, and/or *MiIDAs*-complemented lines were fixed with FAA (10% (*v*/*v*) formaldehyde, 5% (*v*/*v*) acetic acid, 50% (*v*/*v*) ethanol, 35% (*v*/*v*) H_2_O), dehydrated in a gradual ethanol series (50%, 70%, 90%, 95%, and 100%), and critical point-dried (CPD) using Quorum K-850. Next, the samples were coated with gold-palladium (with a Quorum SC7620 mini-sputter coater) and were viewed on a JEOL JCM-6000 benchtop SEM. All analyses were performed with 3 or 4 biological replicates.

### 2.9. Statistical Analysis

Data from different sets of experiments were analyzed using JMP software (JMP Pro14, SAS Institute, Cary, NC, USA). Simple variance analysis (*p* ≤ 0.05) and significance differences were revealed by one-way ANOVA.

## 3. Results

### 3.1. Identification and Isolation of MiIDA1 and MiIDA2 Transcripts from the Mango ‘Kent’ Cultivar

**Figure 1:**
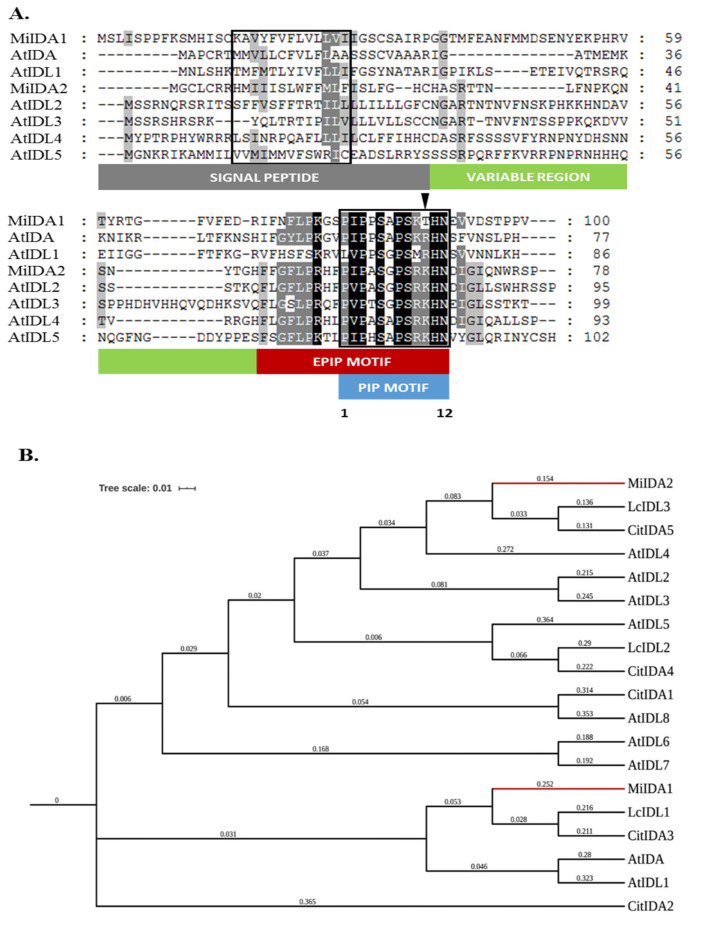
Protein sequence alignment of MiIDAs with Arabidopsis IDA/IDL proteins (**A**), and phylogenetic relationships between MiIDA and other IDA proteins (**B**). (**A**) Alignment was performed using the ClustalW2 program (https://www.genome.jp/tools-bin/clustalw (accessed on 15 August 2018)) with manual adjustment using GeneDoc (MSA editor and shading utility version 2.7.000). The amino acid sequences of MiIDA1 (QGF19396.1), MiIDA2 (QGF19397.1), AtIDA (NM_105550.1), AtIDL1 (NM_113464.2), AtIDL2 (NM_001085327.2), AtIDL3 (NM_001085091.1), AtIDL4 (NM_001084711.1) and AtIDL5 (AY642386.1) were aligned. The signal peptide, the variable region of the extended PIP domain (EPIP) and conserved PIP domain are marked with rectangles. The arrowhead indicates residues at position 10 of the PIP domain. (**B**) Phylogenetic relationships among the mango, lychee, citrus and Arabidopsis IDA proteins. MiIDA1, MiIDA2 LcIDL1 (Lychee_GLEAN_10054315), LcIDL2 (Lychee_GLEAN_10027620), LcIDL3 (Lychee_GLEAN_10009234), AtIDA, AtIDL1, AtIDL2, AtIDL3, AtIDL4, AtIDL5, AtIDL6 (NM_120612.1), AtIDL7 (AK118348.1), AtIDL8 (AK221754.1), CitIDA1 (Ciclev10017342m), CitIDA2 (Ciclev10033211m), CitIDA3 (Ciclev10033191m), CitIDA4 (Ciclev10003011m), and CitIDA5 (Ciclev10026873m) sequences were used. Bootstrap consensus trees were inferred from 100 replicates and graphically designed with iTOL (Interactive tree of life. V5) https://itol.embl.de/ (accessed on 15 August 2018). Branch lengths are indicated.

To identify mango *IDA-like* genes, a BLAST search was performed on a ‘Kent’ transcriptome database. Two genes putatively encoding for IDL proteins were identified, and the corresponding genes were subsequently cloned by RT-PCR (see Material and Methods). The clones obtained, *MiIDA1* and *MiIDA2*, contain open reading frames (ORFs) encoding 101 and 79 amino acid (aa)-long proteins, respectively. As presented in Figure 1A, alignment of the two putative MiIDA sequences with those of the AtIDA and AtIDL1-5 proteins showed that the two mango IDA-like proteins possess a defined predicted N-terminal signal peptide, followed by a variable region and a typical C-terminal region (Extended PIP) containing the 12-residue-long PIP domain [19,20]. Within the conserved PIP domain, both MiIDAs contain six invariant residues, namely, proline at positions 3 and 7, serine at positions 5 and 8, and histidine and asparagine at positions 11 and 12, respectively, together with two residues at positions 2 and 6, representing conservative substitutions (Figure 1A). A closer examination of the MiIDA PIP domains revealed that MiIDA1 shares the highest similarity with the PIP motif of AtIDA/IDL1 proteins, yet unexpectedly it contains a threonine at position 10, instead of the conserved arginine [19,20], thus representing an atypical PIP_T_-type version of IDA/IDL1 proteins. MiIDA2, on the other hand, possesses the typical PIP_K_-type domain found in AtIDL2-5 proteins.

The identity of the two MiIDAs was also confirmed by a phylogenetic analysis. According to the phylogenetic tree presented in Figure 1B, MiIDA1 clustered with AtIDA and AtIDL1, as well as with CitIDA3 and LcIDL1, both suggested to act as key regulators controlling plant organ abscission in citrus trees and lychee, respectively [23,24]. In the same analysis, MiIDA2 clustered with a second eudicots clade of IDL proteins. Taken together, the structural similarity of the two mango MiIDAs to various IDA/IDL proteins, supports the identification of the isolated clones as genes encoding for *IDA-like* proteins. Based on these findings, these genes were registered as *MiIDA1* and *MiIDA2*, accession numbers MN243727.1 and MN243728.1, respectively, in GenBank.

### 3.2. An Atypical PIP_T_-Type Version of ‘Kent’ MiIDA1 Protein Is Present in Various Mango Cultivars

The identification of an atypical PIP_T_-type IDL protein in the ‘Kent’ mango cultivar was unexpected. To test whether this atypical PIP_T_-type version of IDL protein is conserved among other mango cultivars, we used the *MiIDA1* sequence as a query to search for similar *MiIDA1*-encoding genes in the published mango transcriptomes and in EST databases. This led to identifying three independently annotated ESTs from ‘Shelly’, ‘Keitt’, and ‘Langra’ mango cultivars. Examination of the translated products of these ESTs revealed that they all share 100% homology with the translated version of MiIDA1 from the ‘Kent’ cultivar, thus confirming the existence of threonine instead of arginine at position 10 of the mango IDA-like PIP domain (Supplementary Figure S1).

### 3.3. Monitoring the Effect of Ethephon Treatment on the Expression Patterns of MiIDA1 and MiIDA2, and Distinct Ethylene Signaling-Related Genes

**Figure 2:**
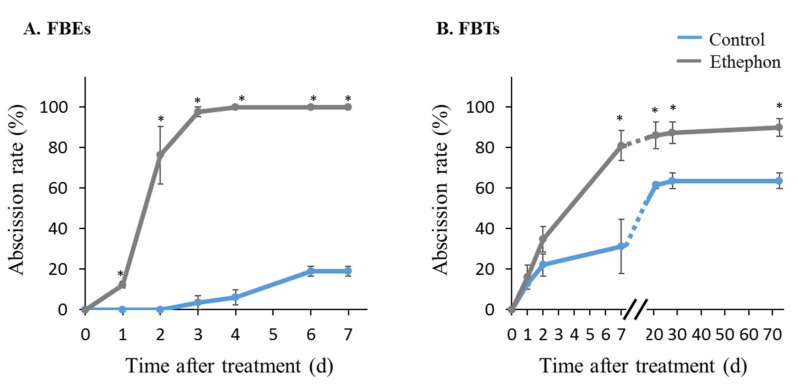
Effect of ethephon treatment on rates of mango fruitlet abscission in fruitlet-bearing explants (FBE) (**A**) and fruitlet-bearing trees (FBT) (**B**). (**A**) Fruitlet abscission was induced with ethephon using three experimental units of FBE, each composed of 110–120 explants. Three untreated experimental units served as controls. The number of intact fruitlets in each experimental unit was monitored at several time intervals, and abscission rates were calculated. Values represent the means + SE of three biological replicates. (**B**) Four trees were sprayed with ethephon to induce abscission, and four control trees were sprayed with surfactant alone. The number of intact fruitlets in each tagged panicle was monitored at several time intervals, and abscission rates were calculated. Values represent means + SE of 60 counts (15 panicles X 4 trees per treatment). Asterisks (*) denote significant differences between ethephon-treated and control explants or trees at each sampling point (*p* ≤ 0.05).

In mango, fruitlet/fruit shedding occurs following the fruit set, during the mid-season, and just before fruit maturity [29]. Our previous records indicated that under north Israel growth conditions, abscission of ‘Kent’ fruitlets during the mid-season reached their highest level at the end of May [34]. To explore a possible association of the cloned *MiIDA* genes with mango fruitlet drop, we conducted experiments at the beginning of May, using both ‘Kent’ FBEs and FBTs. Fruitlet drop was induced by ethephon in both experimental systems (Figure 2). In the case of FBEs, ethephon treatment-induced fruitlet drop within one day. The rate of abscission increased from this time point, reaching almost 100% at three days post-treatment. In contrast, in control explants, fruitlet drop reached only 20% by the end of the observation period at day 7 (Figure 2A). In parallel, the data collected from the field experiment indicated that in FBTs, the rate of abscission reached almost 35% two days after treatment, 81% within seven days, and almost 90% as the fruit reached maturity. By comparison, control trees displayed significantly less, albeit still pronounced, fruitlet drop rates, reaching 62% at the end of the examination period (73 days after treatment), just before harvest (Figure 2B).

**Figure 3:**
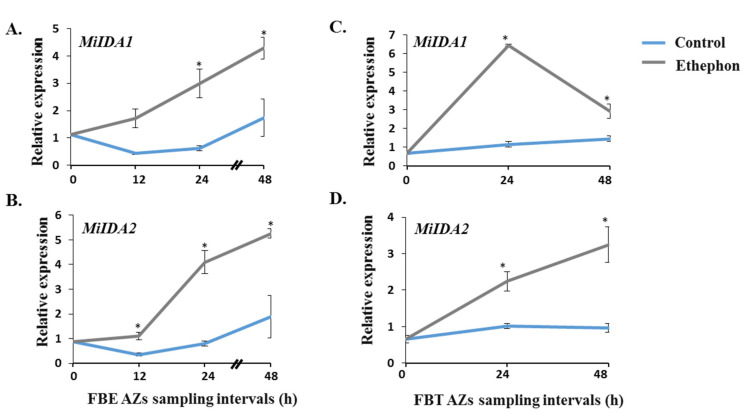
Effect of ethephon treatment on the relative expression of *MiIDA1* and *MiIDA2* genes in mango fruitlet AZs of FBE (**A**,**B**) and FBT (**C**,**D**). Analysis of qRT-PCR was performed using cDNA prepared from total RNA extracted from FBE or FBT AZ tissues as detailed in Materials and Methods. Values represent relative expression levels normalized to that of the endogenous reference gene, *MiGAPDH*. For each group and time point, the data are presented as the means ± SE of three independent biological replicates, and two technical replicates. Asterisks (*) denote significant differences between ethephon-treated and control AZ samples at each sampling point (*p* ≤ 0.05).

qRT-PCR analyses were next carried out using control and ethephon-treated fruitlet AZs, collected at different time points prior to the documentation of abscission rate peaks. Figure 3A,B show that in FBEs, *MiIDAs* transcript accumulation increased following ethephon treatment, and became significantly higher than what was noted in controls within 12 and 24 h for *MiIDA2* and *MiIDA1,,* respectively. The parallel analysis of FBT pedicel AZs showed that over the examined period, the levels of both *MiIDAs* barely increased in control trees (Figure 3C,D). However, following ethephon treatment, *MiIDA1* sharply and significantly increased after 24 h, and then decreased to some extent after 48 h (Figure 3C), while *MiIDA2* transcription exhibited a marked pattern of upregulation starting within 24 h of ethephon treatment (Figure 3D).

**Figure 4:**
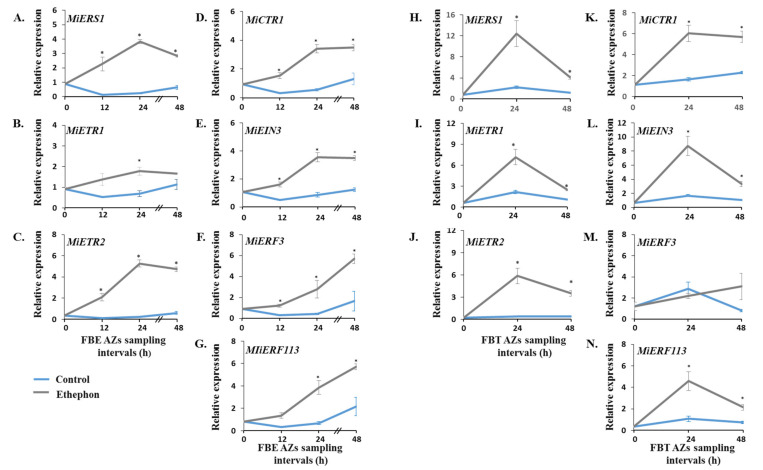
Effect of ethephon treatment on the expression profiles of seven ethylene signaling-related genes in mango fruitlet AZs of FBE (**A**–**G**) and FBT (**H**–**N**) during 48 h. Analysis of qRT-PCR was performed using cDNA prepared from total RNA extracted from FBE or FBT AZ tissues. Values represent relative expression levels normalized against the endogenous reference gene, *MiGAPDH*. For each group and time point, the data are presented as the means ± SE of three independent biological replicates, and two technical replicates. Asterisks (*) denote significant differences between ethephon-treated and control AZ samples at each sampling point (*p* ≤ 0.05).

Ethylene is perceived through its binding to its receptors, which act as negative regulators [38,39,40]. As mentioned above, the expression profiles of *MiERS1* and *MiETR1* ethylene receptors were previously characterized during mango fruitlet drop [31,32,33]. Here, we expanded these studies to examine the effect of ethephon treatment on the expression profiles of additional ethylene signaling-related genes in fruitlet AZs. The corresponding cDNA samples were used to monitor the expression profiles of genes encoding for the ETR2-type ethylene receptor, for the CTR1 kinase, that acts downstream of the ethylene receptors, for the EIN3 ethylene regulator, and for two ethylene responsive factors (ERFs). The expression analyses showed that the ethephon treatment upregulated and/or transiently induced *MiERS1* and *MiETR1* expression within 12–48 h in the AZs of FBE (Figure 4A,B) and FBT (Figure 4H,I), compared to their low expression levels in control tissues. Notably, however, in control FBE AZs, their levels initially slightly decreased and then increased back, whereas in FBT AZs, their levels moderately increased within 24 h. With respect to *MiETR2* (Figure 4C,J), *MiCTR1* (Figure 4D,K), and *MiEIN3* (Figure 4E,L), the AZs assay results showed similar rapid and strong ethephon-induction patterns for the three genes in both systems. Finally, when comparing the two *MiERF* transcripts examined, the expression analysis showed that *MiERF3* and *MiERF113* levels were significantly induced by ethephon within 12–24 h in FBE AZs (Figure 4F,G). On the other hand, in FBT AZs, only *MiERF113* level was significantly induced by ethephon within 24 h and declined later on (Figure 4N).

### 3.4. Monitoring the Effect of Ethephon Treatment on the Expression Profiles of Genes Encoding for Distinct Cell Wall-Modifying Proteins

**Figure 5:**
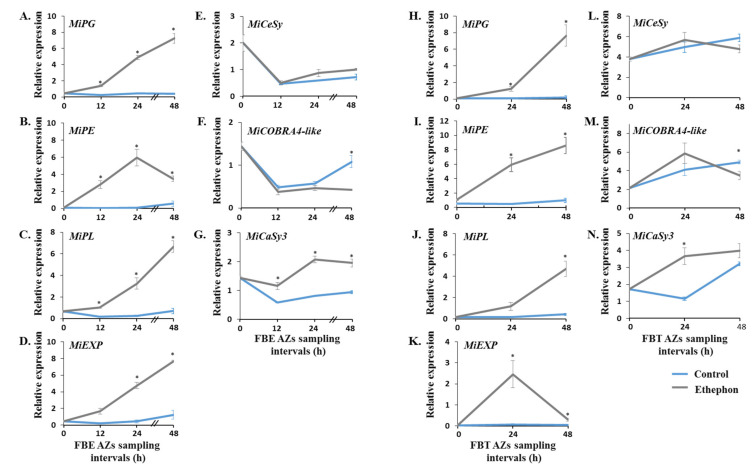
Effect of ethephon treatment on the expression profiles of seven genes encoding for distinct cell wall-modifying proteins in mango fruitlet AZs of FBE (**A**–**G**) and FBT (**H**–**N**) during 48 h. The analysis of qRT-PCR was performed using cDNA prepared from total RNA extracted from FBE or FBT AZs. Values represent relative expression levels normalized against the endogenous reference gene, *MiGAPDH*. For each group and time point, the data are presented as the means ± SE of three independent biological replicates, and two technical replicates. Asterisks (*) denote significant differences between ethephon-treated and control AZ samples at each sampling point (*p* ≤ 0.05).

In this study, we also examined the effect of ethephon treatment on the expression profiles of three pectinonlytic enzyme encoding-genes, namely, genes encoding polygalacturonase (*MiPG*), pectin esterase (*MiPE*) and pectate lyase (*MiPL*), and on *MiEXP* encoding for the cell wall-loosening protein expansin, in fruitlet AZ tissues. The results showed that in control tissues, the relative expression levels of these four genes remained low for 48 h in both FBE (Figure 5A–D) and FBT (Figure 5H–K) pedicel AZs. By contrast, following ethephon treatment, and coinciding with changes observed in ethephon-induced *MiIDA2* transcript levels, *MiPG, MiPE*, and *MiPL* transcript levels were significantly upregulated within 24 h in FBE fruitlet AZs (Figure 5A–C), and within 24–48 h in FBT fruitlet AZs (Figure 5H–J). In the case of *MiEXP*, transcript accumulation was sharply induced by ethephon within 24 h of treatment in FBE pedicel AZs (Figure 5D), whereas in FBT pedicel AZs, a strong initial accumulation level was observed within 24 h, followed by decreased expression within 48 h (Figure 5K). This expression pattern coincides with that observed for *MiIDA1* (Figure 3A,C).

Induction of secondary cell wall-related genes might be required in the last phase of abscission, when the formation of a protective layer at the AZ of the remaining “mother-tree” occurs [3,4,5]. Accordingly, we examined the effects of ethephon treatment on the expression patterns of two secondary cell wall-related transcripts, *MiCeSy* and *MiCOBRA4-like*, putatively encoding for proteins that participate in cellulose synthesis and assembly, respectively. We also monitored the expression profile on *MiCaSy3,* encoding for callose synthase, a protein that might function at sites where tissues have been damaged. In AZs collected from both control and ethephon-treated FBEs, the levels of *MiCeSy* decreased within 12 h and remained low for up to 48 h of the examination period (Figure 5E). A similar expression pattern was observed for the *MiCOBRA4-like* gene, except that within 24–48 h, its expression level was restored to its original level in the control tissue (Figure 5F). In FBT AZs, *MiCeSy* and *MiCOBRA4-like* expression levels remained relatively stable over the period examined, and hardly differed between control and ethephon-treated tissues (Figure 5L,M). The *MiCaSy3* gene revealed similar expression patterns in fruitlet pedicel AZs sampled from both FBEs and FBTs. Specifically, *MiCaSy3* expression levels slightly decreased within 12 h in control FBE (Figure 5G), or within 24 h in control FBT (Figure 5N), and then increased within 48 h in both control tissues. By contrast, in ethephon-treated AZ tissues of both FBE and FBT, *MiCaSy3* expression levels markedly increased within 24 h, and remained high until the end of the examination period (Figure 5G,N).

### 3.5. Overexpression of MiIDA1 and MiIDA2 in Arabidopsis Promoted Floral Organ Abscission, and Restored Floral Organ Abscission Ability in an Atida Mutant

**Figure 6:**
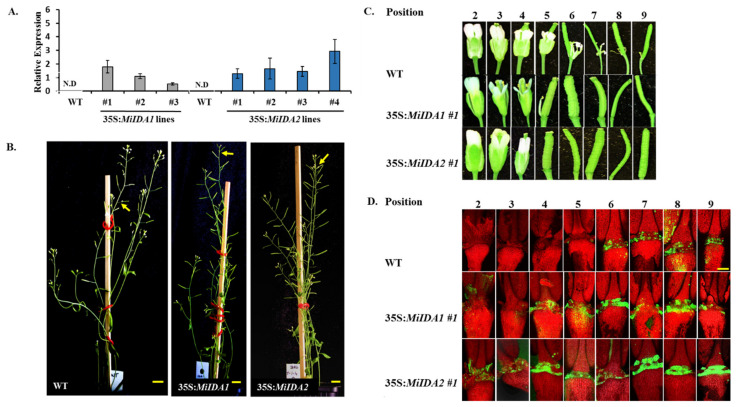
Ectopic expression of *MiIDA1* and *MiIDA2* in Arabidopsis promotes early floral organ abscission. (**A**) Relative expression levels of *MiIDA1* (grey columns) and *MiIDA2* (blue columns) in WT, 35S:*MiIDA1*, and 35S:*MiIDA2* transgenic Arabidopsis plants. Values represent relative expression levels normalized against the *AtACT* gene, and the data are presented as means + SE of four replicates per line. ND, not detected. (**B**) Phenotype comparison of Arabidopsis Col-0 (WT) and two representative overexpressing 35S:*MiIDA1* and 35S:*MiIDA2* lines. Scale bar = 1 cm. Arrows denote attached floral parts. (**C**) Phenotypes of floral organ abscission in Arabidopsis Col-0 (WT) and in 35S:*MiIDA1-* and 35S:*MiIDA2*-overexpressing plants. Flowers and siliques from different positions along the inflorescences were sampled separately from WT, and the 35S:*MiIDA1* and 35S:*MiIDA2* lines. Flower position numbers are indicated starting from the second flower at anthesis. The images presented for each WT plant or overexpressing lines are representative images of 3 or 4 replicates. (**D**) Representative BCECF fluorescence micrographs of floral organ AZs of Arabidopsis Col-0 (WT), and 35S:*MiIDA1* and 35S:*MiIDA2* plants. Intact flowers and siliques were sampled separately, incubated in BCECF solution, and examined with a confocal laser scanning microscope. Images represent merged images of BCECF fluorescence with chlorophyll auto-fluorescence images. The green color represents the increase in pH (detected by increased BCECF fluorescence), and the red color represents chlorophyll auto-fluorescence. Flower position numbers are indicated, starting from the second flower at anthesis. Scale bar = 200 µm. The images presented for each WT plant or overexpression line are representative images from 3 or 4 replicates.

To assess whether *MiIDA1* and *MiIDA2* promote floral organ abscission in a similar manner as does the *AtIDA*, cDNAs of the *MiIDA* genes were initially expressed in WT Arabidopsis Col-0 plants, driven by the constitutive cauliflower mosaic virus (CaMV) 35S promoter. Following transformation, the F_2_ progeny of three 35S:*MiIDA1* and four 35S:*MiIDA2* randomly selected lines, were subjected to detailed phenotypic analysis. Analysis by qRT-PCR first corroborated that *MiIDA1* and *MiIDA2* transcripts were expressed in the corresponding transformed Arabidopsis plants (Figure 6A). Analysis of mature plant heights revealed that both *MiIDA1-* and *MiIDA2*-overexpressing lines were slightly shorter, in comparison to WT plants (Supplementary Figure S2A). On the other hand, the mature silique lengths of the transformed lines did not significantly differ from those of WT plants (Supplementary Figure S2B). Further examination of flower parts/organs at different positions along the inflorescences showed that both *MiIDA1* and *MiIDA2* overexpressing plants exhibited early drop of floral parts/organs, as compared to WT plants (Figure 6B,C). Knowing that abscission of flower organs in Arabidopsis is associated with alkalization of the cytosol in AZ cells [37], we used the pH-sensitive BCECF dye to monitor pH changes in floral AZ cells of the 35S:*MiIDA1-* and 35S:*MiIDA2-*overexpressing plants as compared to WT plants. Confocal microscopy images showed that in WT plants, an increase in green fluorescence, mirroring an increase in cytosolic pH values, started in floral AZs at position (P) 6, and was augmented from P6 to P9 (Figure 6D and Supplementary Figure S2C). By contrast, an increase in the green fluorescence in the 35S:*MiIDA1* and 35S:*MiIDA2* floral AZs started much earlier at P2 and P3 (Figure 6D), thereby confirming that both *MiIDA1* and *MiIDA2* promote early floral organ abscission (Figure 6C). Interestingly, in the overexpressed *MiIDA* lines, the increase in the green fluorescence was observed already at P2 (Figure 6D), before the drop of their flower organs started at P5 (Figure 6C), while in WT plants the appearance of the green fluorescence occurred in parallel to the drop of floral organs at the same position (P6) (Figure 6C,D).

**Figure 7:**
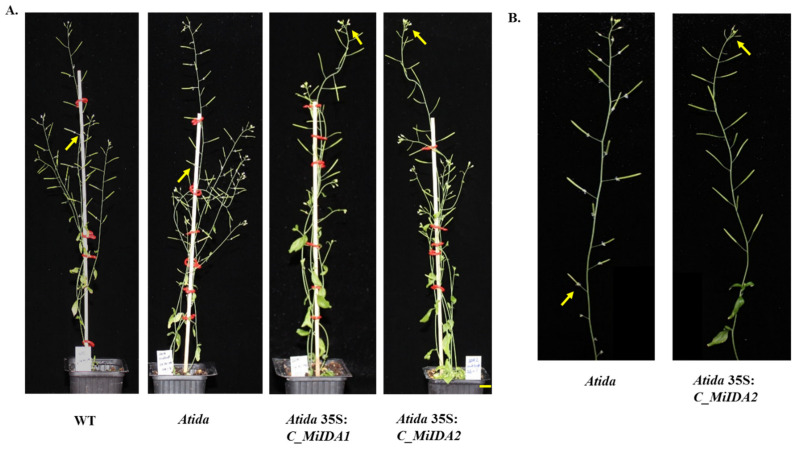
*MiIDA1* and *MiIDA2*, expressed under control of the 35S promoter, complemented and restored floral organ abscission ability in an *Atida* mutant. Phenotypes of fully developed WT, *Atida* mutant, *Atida* 35S:*C_MiIDA1-* and *Atida* 35S:*C_MiIDA2*-complemented plants (**A**), and enlarged photos of the *Atida* mutant and *Atida* 35S:*C_MiIDA2*-complemented plants (**B**), are presented. Scale bar = 1 cm. Arrows denote attached floral parts.

**Figure 8:**
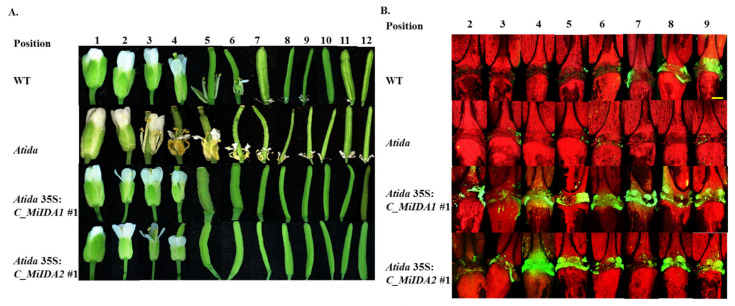
MiIDA1 and MiIDA2 complemented and restored floral organ abscission ability in an *Atida* mutant. (**A**) Comparison of phenotypes of floral organ abscission in WT, *Atida* mutant, *Atida* 35S:*C_MiIDA1*, and *Atida* 35S:*C_MiIDA2* plants. (**B**) Representative BCECF fluorescence micrographs of floral organ AZs of Arabidopsis Col-0 (WT), an *Atida* mutant, and *Atida* 35S:C *MiIDA*-complemented plants. Flower position numbers are indicated, starting from the second flower at anthesis. Scale bar = 200 µm. The images presented for each plant type (WT, mutant or complemented plants) are representative images of 3 or 4 replicates.

To further test whether *MiIDA1* and *MiIDA2* transcripts could complement AtIDA function, we generated transgenic plants expressing 35S:*MiIDA1* and 35S:*MiIDA2* in an *Atida* mutant background. As in the case of the *MiIDA-*overexpressing plants, RT-PCR analysis confirmed that *MiIDA1* and *MiIDA2* transcripts were expressed in the corresponding complemented plants (Supplementary Figure S3). Phenotypic examination demonstrated that, as expected, *Atida* mutant plants were deficient in their ability to abscise their floral organs, as compared to WT plants (Figure 7 and Figure 8). Furthermore, a closer examination revealed that in WT plants, floral organs abscission started at P6-P7, and the sepals, petals, and stamens remained turgid up to P4-P5 (Figure 8A). By comparison, in the *Atida* mutant plants, browning and dehydration of the floral parts started from P3, and the stamens remained dehydrated and attached to the plant body up to P12 (Figure 8A). On the other hand, a strong restoration of the abscission ability of the floral organs was observed in *Atida* 35S:*C_MiIDA1* and *Atida* 35S:*C_MiIDA2* plants (Figure 7). As such, it was observed that the floral organs of *Atida* 35S:*C_MiIDA1*- and *Atida* 35S:*C_MiIDA2*-complemented plants dropped early at P5, displaying turgid sepals and petals up to P4 (Figure 8A). Lastly, in line with these observations, a strong BCECF green fluorescent signal could be detected in *Atida* 35S:*C_MiIDA1* and *Atida* 35S:*C_MiIDA2* floral AZ cells already at P2, much earlier than detected in WT plants (at P6), whereas, in the *Atida* mutant, BCECF green fluorescence could be hardly detected up to P9 (Figure 8B). Furthermore, coinciding with *MiIDAs* overexpressing lines BCECF results (Figure 6D), in *MiIDAs* complemented plants, an increase in the BCECF green fluorescence signal could be detected at three positions before the drop of their flower organs started (Figure 8).

### 3.6. Overexpression of MiIDA1 and MiIDA2 in Arabidopsis Is also Associated with Activation of Cell Separation at the Base of Pedicel AZs

**Figure 9:**
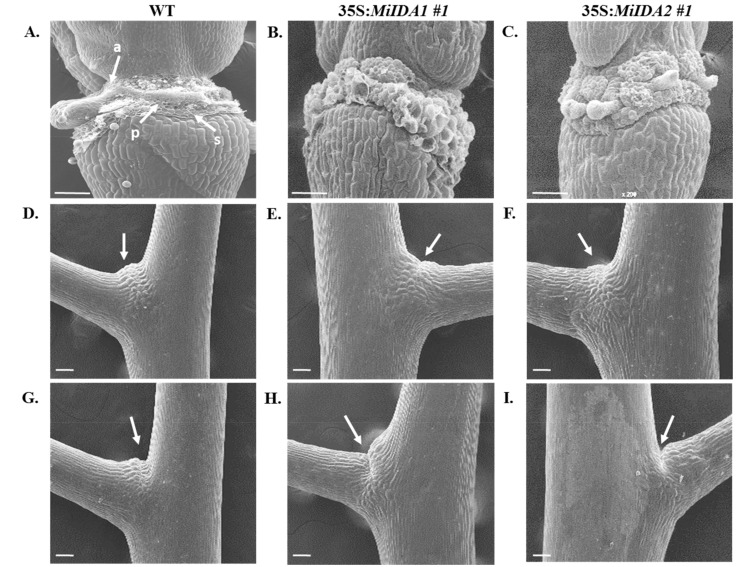
Scanning electron micrographs of floral and pedicel vestigial AZs. Floral parts after abscission at the AZs of (**A**) WT, (**B**) 35S:*MiIDA1,* and (**C**) 35S:*MiIDA2*. AZ sites of sepals (s), petals (p), and anthers (a) are denoted. (**D**) WT, (**E**) 35S:*MiIDA1* and (**F**) 35S:*MiIDA2* young (P5-P7) pedicel vestigial AZs. Arrows denote a small cleft developed on the upper side of the pedicel. (**G**) WT, (**H**) 35S:*MiIDA1* and (**I**) 35S:*MiIDA2* old (P8-P10) pedicel vestigial AZs. Arrows denote an invasive developed cleft on the upper side of the pedicel. All scale bars = 100 µm. The images presented for each WT plant or overexpression line are representative images of 3 or 4 replicates.

Arabidopsis studies demonstrated that overexpression of *AtIDA* driven by the 35S promoter enhanced cell separation not only in the AZs of floral organs, but also in other vestigial AZs [10]. To test whether a similar phenomenon occurs in the 35S:*MiIDA1* and 35S:*MiIDA2* lines, floral and vestigial AZs (found at the base of the pedicels) were examined by SEM, relative to the same tissues in WT control plants. As reported by Stenvik et al. [10], we noted that after the abscission of floral organs took place in the WT plants, the AZ area exhibited rounded cells, and those positions where the shedding of the different floral organs (sepals, petals, and filaments) had occurred, were clearly visible (Figure 9A). By comparison, 35S:*MiIDA1* and 35S:*MiIDA2* floral AZs displayed much more prominent rounded cells with substantially larger rounded dead cells, which engulfed the whole AZ fracture plane (Figure 9B,C). Examination of the AZs located at the base of the pedicels showed that in WT plants (Figure 9D), as well as in 35S:*MiIDA1* and 35S:*MiIDA2* plants (Figure 9E,F), a small cleft appeared on the upper side of younger pedicels at P5-P7. A more invasive cleft appeared in the AZs of older pedicels at P8-P10 (Figure 9G–I), suggesting that degradation of the middle lamella had occurred. This was, however, much more prominent in the base of older pedicels of 35S:*MiIDA1* and 35S:*MiIDA2* plants (Figure 9H,I) than in older pedicel samples of control WT plants (Figure 9G). These observations provide additional evidence that abscission events occur earlier in the *MiIDA*-overexpressing lines than in the WT control plants.

## 4. Discussion

Premature fruitlet/fruit abscission has a major impact on the crop productivity of tropical and sub-tropical fruit trees, including that of mango [28]. The mechanism(s) controlling mango fruitlet/fruit drop are not fully understood, making it difficult to develop approaches to solve this problem. Earlier studies in Arabidopsis identified the importance of the IDA-HAE/HSL2 module in controlling floral organ abscission [9,10,11]. Recent studies suggest that proteins similar to AtIDA are conserved among different plant species, including fruit trees, such as citrus, oil palm, and lychee, although the specific mode of action of the IDA-like proteins in these crop species is largely unknown [19,23,24,25]. Moreover, up to date, no information regarding *IDA*-*like* encoding genes from mango was available.

In the present study, we isolated two *IDA-like* genes from the ‘Kent’ mango cultivar. Based on amino acid sequence alignments, we confirmed that both MiIDA1 and MiIDA2 contain elements, which are present in IDA/IDL proteins, including defined N-terminal signal peptides (Figure 1A), putatively targeting the proteins to the apoplast, and defined PIP domains [18,19]. The isolation of *MiIDA1* and *MiIDA2* genes allowed us to investigate their expression profiles in fruitlet pedicel AZs before the onset of enhanced fruitlet drop induced by ethephon. In FBE AZs, a significant ethephon-induction of *MiIDAs* transcripts, as compared to control, was depicted already within 12–24 h (*MiIDA2* and *MiIDA1*, respectively). In FBT AZs, the levels of both genes sharply increased, as compared to control, within 24 h. Concomitant with the upregulation of *MiIDA1* and *MiIDA2*, ethephon-treated AZs also displayed pronounced upregulation of the ethylene receptor genes, *MiERS1* and *MiETR1* (Figure 4). In line with our and others’ earlier findings, a stronger response was observed for *MiERS1*, as compared to *MiETR1* [31,32]. Furthermore, we were also able to detect a rapid ethephon-mediated expression induction of *MiETR2, MiCTR1*, and *MiEIN3*, which was accompanied by upregulation of an ethylene responsive factor transcript, *MiERF113* (Figure 4), indicating that ethylene levels in the AZ likely increased, and that ethylene was perceived in these tissues.

It should be mentioned that initial studies of AtIDA proposed that this small, secreted protein controls floral organ abscission via an ethylene-independent pathway, leading to the activation of cell wall-modifying genes [9,14]. On the other hand, it was later reported that ethylene could induce *IDA-like* and/or *HSL-like* gene expression in AZ tissues of lychee and lupine flowers, as well as in oil palm fruit AZ, prior to the start of organ abscission [19,24,26]. Ethylene was also shown to induce the expression of *IDA-like* genes from tomato and soybean, whereas treatment with the ethylene action inhibitor 2,5-norbornadiene delayed the increase in the expression of these genes [22]. These and other findings led to establishing a different concept, suggesting that the IDA/IDL pathway is actually an ethylene-dependent abscission module, which operates downstream of ethylene, serving as a signal for late and/or post-abscission events [15,16]. In agreement with this concept, our results reinforce the model suggesting that the IDA/IDL pathway operates in an ethylene-dependent manner in mango.

Our observations, thus, fit a scenario whereby following ethephon treatment (inducing ethylene release and subsequent responses), upregulation of both *MiIDA1* and *MiIDA2* transcripts affect mango fruitlet drop. The question that remains to be deciphered is related to the identity of the endogenous genes controlled by *MiIDA1* and *MiIDA2*. The emerging concept from early Arabidopsis studies was that the IDA-HAE/HSL2 module is responsible for transcriptional activation of cell wall degradation and cell-wall loosening-related genes, thereby leading to organ abscission [12]. However, based on a subsequent re-evaluation of microarray data and RNA-seq analysis, it was suggested that the AtIDA-HAE/HSL2 module regulates only a subset of specific cell wall-modifying genes [41,42,43]. Notably, in the case studied here, in both our experimental mango systems, ethephon-mediated induction of *MiIDA2* transcripts (Figure 3) was accompanied by upregulation of *MiPG*, *MiPE*, *MiPL*, and *MiCaSy* transcript levels, while upregulation of *MiIDA1* was associated with changes in *MiEXP* expression profiles (Figure 5). Our results might suggest that, as proposed for other model organisms [12], *MiIDAs* control mango fruitlet abscission by affecting the expression of cell wall-remodeling genes, such as those monitored here. Alternatively, these results can be interpreted as representing a direct effect of ethylene on the expression of these cell wall-related genes. Indeed, an ethylene-mediated induction of cell wall-remodeling genes at AZs prior to fruitlet abscission events, is well documented [1,6]. With this in mind, any conclusions regarding the mode action of MiIDA1 and MiIDA2 in regulating mango fruitlet abscission should be drawn with caution.

Furthermore, phylogenetic analysis showed that MiIDA1, in particular, is closely related to the small group of PIP_R_-IDA/IDL proteins associated with abscission events, despite containing an atypical PIP_T_ domain (Figure 1B). At the same time, MiIDA2 clusters with members of a second PIP_K_-IDA/IDL family of proteins, that were proven to substitute IDA function to varying degrees [9,18]. Given the observation that ‘Kent’ *MiIDA1* encodes for a protein that contains an atypical PIP_T_ domain instead of the conserved PIP_R_ domain [19,20], it was of special interest to examine whether this atypical form of the *IDL* gene is also present in other mango cultivars. Indeed, the existence of an atypical PIP_T_-type version of *IDL* in mango was confirmed with the detection of three independently annotated mango ESTs from three additional mango cultivars, all putatively encoding for MiIDA1-like proteins with a PIP_T_-domain (Supplementary Figure S1). In this context, it is important to note that Arabidopsis studies have shown that overexpression of PIP_R_-type *AtIDA* under the constitutive 35S promoter resulted in earlier abscission of floral parts, as well as in abscission events at the base of the pedicels, branches, and cauline leaves [10,18]. Moreover, the PIP_k_-type of 35S:*AtIDL2-5* overexpressing plants displayed similar phenotypes as that of 35S:*AtIDA* [18]. Since *MiIDA1* encodes for a protein that contains an atypical PIP_T_ domain instead of the conserved PIP_R_ domain [19,20], it was important to examine whether this form of the *IDA-like* gene functions in a similar mode as does AtIDA, and to test in parallel the ability of MiIDA2 to induce abscission. Interestingly, when examining the ectopic expression of *MiIDA1* and *MiIDA2* in Arabidopsis, we were able to show that transgenic plants overexpressing both mango genes, were characterized by early floral organ abscission, and by an early increase in the cytosolic pH of floral AZ cells (Figure 6), a change shown to be associated with the progress of abscission [24,37]. Moreover, our complementation assays confirmed that both *MiIDA* transcripts could restore the effects of *Atida* deficiency on floral organ shedding (Figure 7 and Figure 8). Lastly, SEM images of mango fruitlet AZs revealed that overexpression of both genes was associated with early abscission of floral parts, reflected by a prominent ‘rounding up’ phenotype of the AZ cells [10], and by activation of cell separation at the base of the pedicel AZ (Figure 9).

As mentioned early, indications that IDA/IDL PIP_R_-IDA proteins from citrus and lychee play similar roles as does AtIDA, were previously provided by studies showing that overexpression of genes encoding for these proteins under control of the 35S promoter (*CiIDA3* and *LcIDL1*, respectively) induced early floral organ abscission [23,24]. Confirmation of the conserved roles of the products of *CiIDA3* and *LcIDL1* genes was also obtained in the *Atida2* mutant complementation assays [23,24]. Our results similarly suggest that the functions of both mango PIP_T_-type-IDA/IDL and PIP_k_-type-IDA/IDL are linked with the induction of abscission events in Arabidopsis.

PIP_R_-AtIDA is suggested to affect floral organ abscission via the AtLRR-RK HAESA (HEA) and HAE-like2 (HSL2) receptors (for review, see [20]). In fact, the crystal structure of a synthetic AtIDA peptide bound to its LRR-RK HAESA receptor was resolved, and demonstrated that a central hydroxyproline residue at position 7 in the AtIDA PIP ligand domain (also present in both MiIDAs) anchors IDA to its receptor [44]. It was also reported that the AtHAESA co-receptor AtSERK1, acting as a positive regulator of the floral abscission pathway, allows the high-affinity sensing of the AtIDA peptide by binding to the arginine, histidine, and asparagine residues (at positions 10, 11, and 12, respectively) of the PIP_R_ domain [44]. Finally, it is important to mention that while at present, receptors of PIP_k_-AtIDL proteins have yet to be identified [20], the finding that overexpression of *AtIDL2-5* under control of the 35S promoter resulted in early floral organ abscission, suggests that PIP_k_ type IDA peptides might also signal through the AtIDA receptor [18]. Note, however, that a high quantity of protein may overshadow the suboptimal peptide-receptor quality of binding, as it turned out that only *AtIDL1* could complement the *ida* mutation under the control of *AtIDA* own promoter [18]. Keeping this notion in mind, the results obtained here indicate that despite the anticipated low quality of binding of MiIDA1 (possessing a PIP_T_-type domain) and of MiIDA2 (possessing a PIP_k_-type domain) to AtHAESA receptors, high quantities of MiIDA1 and MiIDA2 proteins, whose expression was driven by the 35S promoter, can induce abscission.

Lastly, our results might contribute to improve our understanding of distinct roles played by IDA/IDL proteins. For instance, the abscission process is suggested to be associated not only with an increase in cytosolic pH in AZ cells, but also in an increase in reactive oxygen species (ROS) production, which likely facilitates water influx to AZ cells to enable cell expansion [45,46]. The possible importance of aquaporins in this process was previously reviewed in detail [16]. Furthermore, a study from Wang et al. [47], recently demonstrated that induction of the tonoplast intrinsic proteins (TIPs), which belong to the aquaporin family, is important for the tomato flower pedicel abscission. It is also of interest to note that in a current re-evaluation of the role played by AtIDA during abscission, it was suggested that an *ida* mutant, showing a defect in its flower organ abscission, might be a result of IDA signals affecting water relations of the AZ cells via aquaporins [16]. Here, our results showed that while fast dehydration of petals and stamens occurred already at P2-P3 in the *Atida* mutant plants, both S35:*MiIDAs* complemented plants displayed turgid petals at P4, before they abscised (Figure 8A). These observations, together with the increased observed cell ‘rounding up’ phenotype in AZs of the S35:*MiIDAs* overexpression lines (Figure 9B,C), support the idea that IDA/IDL may play an important role in affecting the water balance of AZ cells. Moreover, in this study, we also noted that while an almost null BCECF green fluorescence signal could be detected in *Atida* mutants, a strong and early signal was detected in 35S:*C_MiIDA1* and *Atida* 35S:*C_MiIDA2* floral AZ cells, before the floral organ abscission started at P3-P4 (Figure 8B). Indeed, a similar phenomenon of early detection of the BCECF green fluorescence, prior to cell separation, was observed when the lychee *LcIDL1* was overexpressed in WT Arabidopsis plants or in an *Atida2* mutant background [24]. Together, these results may suggest an additional possible role for IDA/IDL in affecting the cytosolic alkalization of the AZ cells, which precedes the cells separation process.

## 5. Conclusions

Our data provide different lines of evidence, supporting the conserved functions of both MiIDA1 and MiIDA2 in regulating mango fruitlet abscission, and suggest that the putative mango IDA-HAE/HSL2-like module operates in an ethylene-dependent manner. Furthermore, our results indicate that both MiIDA1 and MiIDA2 might function in a similar mode as AtIDA, to promote organ abscission. We propose that upon ectopic expression in Arabidopsis, MiIDA1 and MiIDA2 might be involved in regulating flower organ abscission by affecting aquaporin-encoding genes expression and/or by imposing early cytosolic pH changes in AZ cells.

Given the present findings, further research is now required to identify and characterize the different components and targets of the mango IDA-HAE/HSL2-like module. This is especially true given the identification of an atypical PIP_T_ version of the mango IDA/IDL protein. The recent release of different mango genome sequences [48,49], opens now new avenues to explore the mango IDA-HAE/HSL2-like module. As such, a BLAST search using ‘Tommy Atkins’ mango genome led us to identifying three HAESA-like predicted proteins. Whether these genes function as receptors that interact with MiIDA1 or MiIDA2, remains to be established. We predict that future adaptations of the CRISPR/Cas gene editing technique to mango, generating relevant *MiIDA1* or *MiIDA2* mutants and their corresponding receptors, might help define the endogenous functions of these components, and/or could be used as biotechnological tools to decrease mango fruitlet drop.

## Data Availability

Not applicable.

## Supplementary Materials

The following are available online at https://www.mdpi.com/2073-4425/12/3/439/s1, Figure S1: Predicted amino acid sequences of MiIDA1 proteins from various mango cultivars. The signal peptide, the variable region of the extended PIP domain (EPIP), and the conserved PIP domain, are marked with rectangles. The arrowhead indicates the residues at position 10 of the PIP domain, Figure S2: Comparison between Arabidopsis Col-0 (WT), and 35S:*MiIDA1-* and 35S:*MiIDA2*-overexpressing phenotypes. (A) Averaged main shoot plant lengths. (B) Averaged silique lengths. Values represent means + SE of four measurements per line for main shoot lengths, and of 16 measurements for lengths of siliques from positions 10–14. (C) BCECF fluorescence micrographs of the floral organ AZs of Arabidopsis Col-0 (WT), and distinct independent 35S:*MiIDA1-* and 35S:*MiIDA2-* overexpressing lines. Intact flowers or siliques were sampled separately from WT plants, and from the 35S:*MiIDA1* and 35S:*MiIDA2* independent lines, incubated in BCECF solution, and examined with a confocal laser scanning microscope. Images represent merged images of BCECF fluorescence with chlorophyll auto-fluorescence images. Position numbers are indicated from the second flower at anthesis. Scale bar = 200 μm, Figure S3: Relative expression levels of *MiIDA1* (grey columns), and *MiIDA2* (blue columns) in *Atida* 35S:*C_MiIDA-*complemented plants. Values represent relative expression levels normalized against the *AtACT,* and are means + SE of four replicates per line. ND, not detected, Table S1: Primers used for *MiIDA1* and *MiIDA*2 isolation, construct preparation, and expression analyses in transformed Arabidopsis plants, Table S2: Primers used for mango AZ real-time qPCR analysis.
