# Supplementary material for: Characterization of Two Ethephon-Induced IDA-Like Genes from Mango, and Elucidation of Their Involvement in Regulating Organ Abscission
Source: Genes (Basel). 2021 Mar 19;12(3):439. doi: 10.3390/genes12030439 (PMC8003476; doi:10.3390/genes12030439)
Supplement: Supplementary file 1 [file genes-12-00439-s001.zip › Supplementary files Rai et al 2021.docx]

**
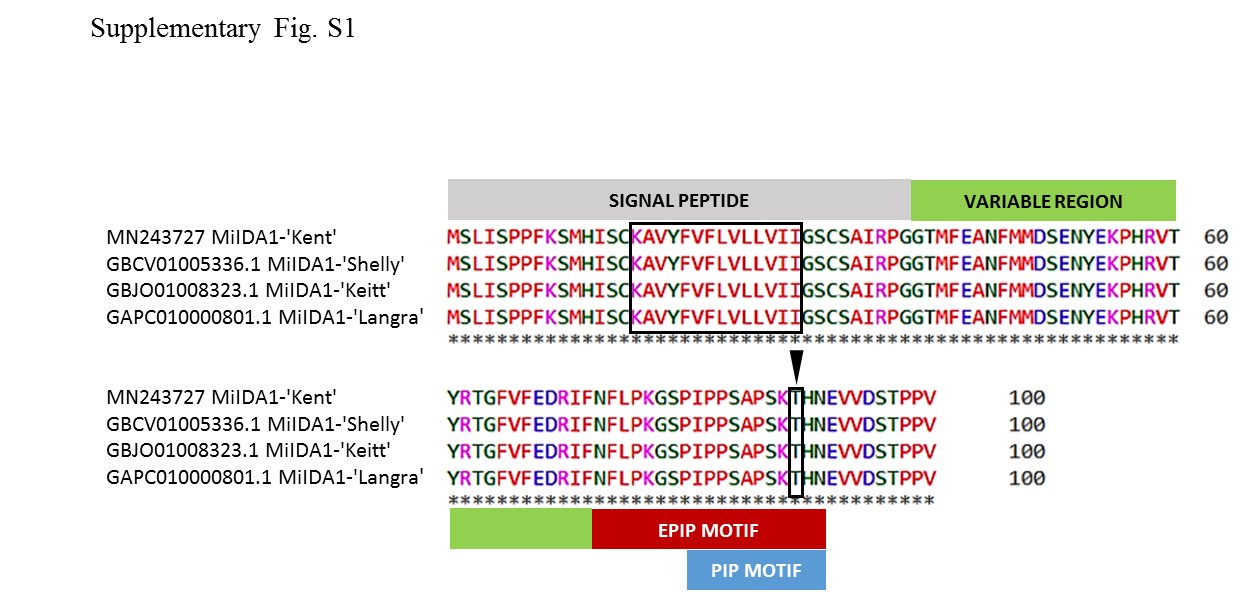
**

**Figure S1**: Predicted amino acid sequences of MiIDA1 proteins from various mango cultivars. The signal peptide, the variable region of the extended PIP domain (EPIP) and the conserved PIP domain, are marked with rectangles. The arrowhead indicates the residues at position 10 of the PIP domain.

**
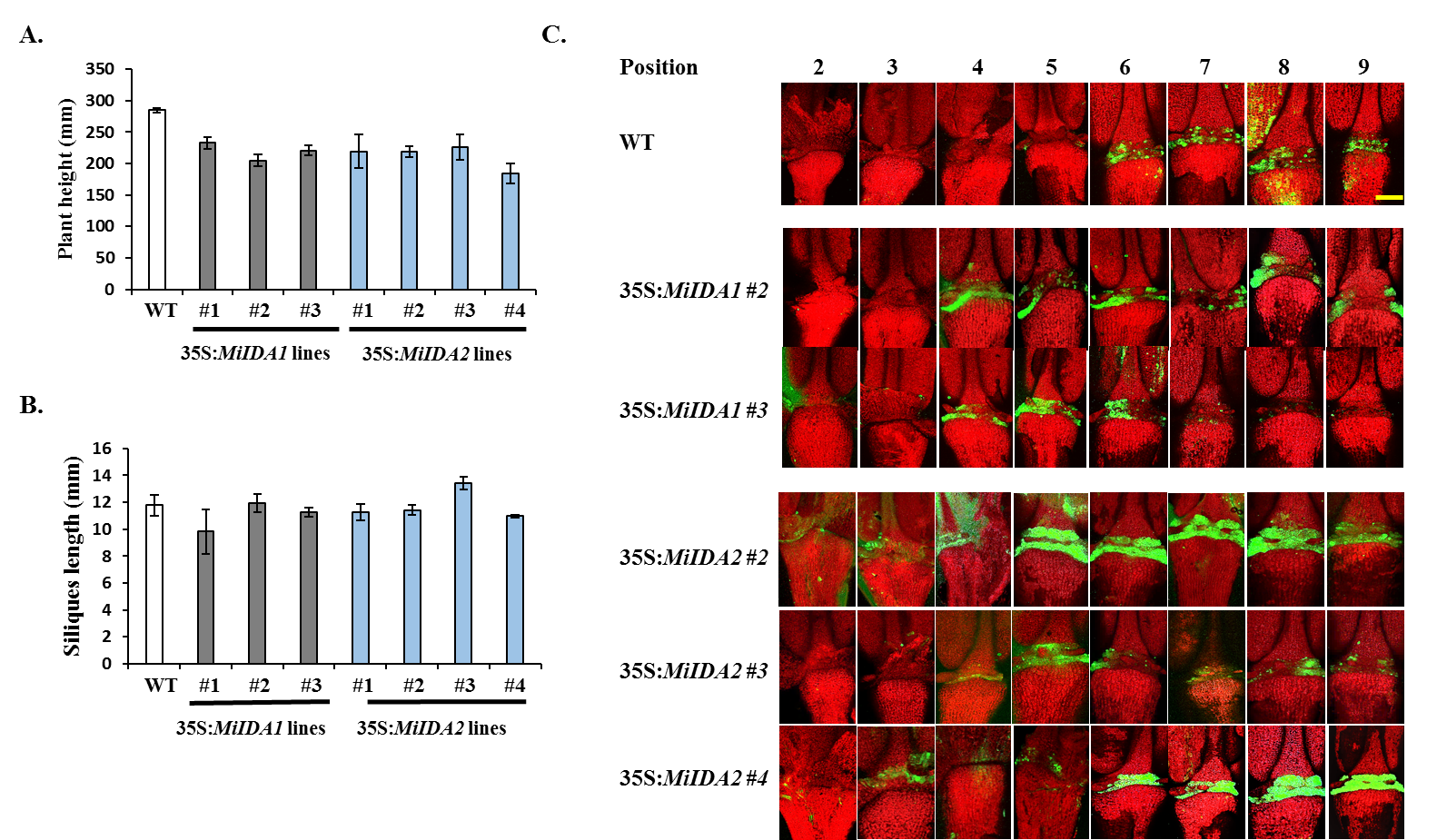
**

**Figure S2:** Comparison between Arabidopsis Col-0 (WT), and 35S:*MiIDA1-* and 35S:*MiIDA2***-**overexpressing phenotypes. (A) Averaged main shoot plant lengths. (B) Averaged silique lengths. Values represent means + SE of four measurments per line for main shoot lengths, and of 16 measurements for lengths of siliques from positions 10-14. (C) BCECF fluorescence micrographs of the floral organ AZs of Arabidopsis Col-0 (WT), and distinct independent 35S:*MiIDA1-* and 35S:*MiIDA2-* overexpressing lines. Intact flowers or siliques were sampled separately from WT plants, and from the 35S:*MiIDA1* and 35S:*MiIDA2* independent lines, incubated in BCECF solution, and examined with a confocal laser scanning microscope. Images represent merged images of BCECF fluorescence with chlorophyll auto-fluorescence images. Position numbers are indicated from the second flower at anthesis. Scale bar = 200 mμ.

**
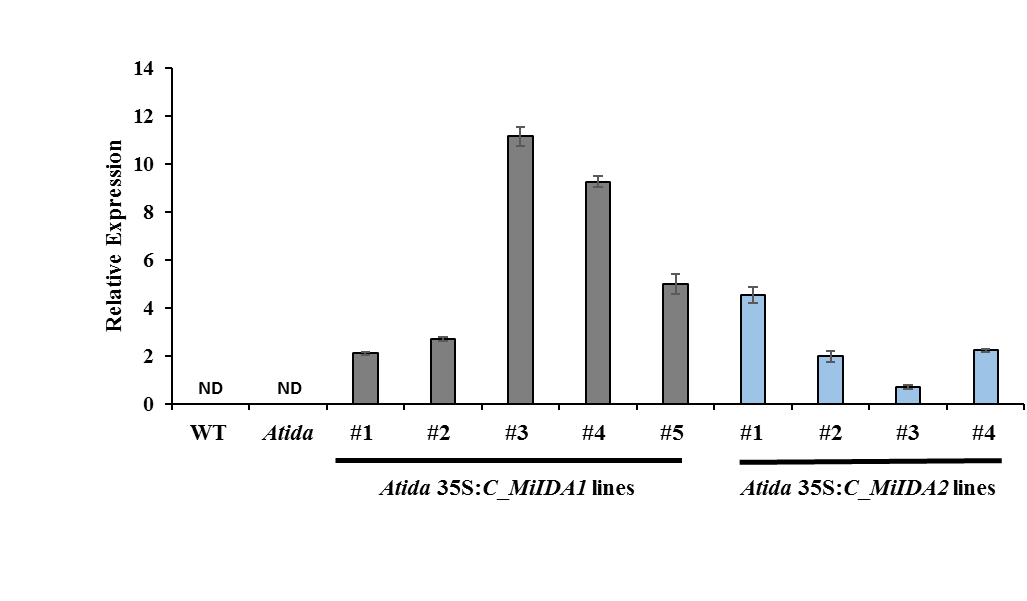
**

**Figure S3**: Relative expression levels of *MiIDA1* (grey columns), and *MiIDA2* (blue columns) in *Atida* 35S:*C_MiIDA-*complemented plants. Values represent relative expression levels normalized against the *AtACT,* and are means + SE of four replicates per line. ND, not detected.

**Table S1:** Primers used for *MiIDA1* and *MiIDA*2 isolation, construct preparation, and expression analyses in transformed Arabidopsis plants.

| **Primer Name** | **Used for** | **Sequence (5'→3')** |
| --- | --- | --- |
| MiIDA1-F- Full | *MiIDA1* full length isolation | GCTTTTCAACTTTCTGCTGCCT |
| MiIDA1-R- Full |  | GCATTGTCATGATCCATTAAGGTGA |
| MiIDA2-F- Full | *MiIDA2* full length isolation | TCCTTAAAGGCTTAAACTCTACCG |
| MiIDA2-R- Full |  | CACTTGAAAAACCTCTGCAA |
| MiIDA1_Xho1_F | pART27 35S:*MiIDA1* construction | TTCTCTCGAGGCTTTTCAACTTTCTGCTGCCT |
| MiIDA1_Xba1_R |  | AAGTTCTAGAGCATTGTCATGATCCATTAAGGTGA |
| MiIDA2_Xho1_F | pART27 35S:*MiIDA2*  construction | TTCTCTCGAGTCCTTAAAGGCTTAAACTCTACCG |
| MiIDA2_Xba1_R |  | AAGTTCTAGACACTTGAAAAACCTCTGCAA |
| 5’UTR_F | *Atida* homozygosity confirmation | TGGCCGTAATGACCTTAAACAT |
| 3’UTR_R |  | CACTCCACTCTTACCATTCCCC |
| LB_R |  | AACTGGAACAACACTCAACCCT |
| AtACT_F | *MiIDAs* RT (reference gene) | TCTTAACCCAAAGGCCAACA |
| AtACT_R |  | CCAGAATCCAGCACAATACC |
| MiIDA1-F-RT | *MiIDA1* RT | GGTCCTTCACGGAAACACAATG |
| MiIDA1-R-RT |  | GGGAAGAAGAAGAAGGGGAAAA |
| MiIDA2-F-RT | *MiIDA2* RT | GGTCTCCTATTCCGCCTTCTG |
| MiIDA2-R-RT |  | CCAATTGAGAGAGAACCGCCTA |

**Table S2:** Primers used for mango AZ real-time qPCR analysis

| **Primer name** | **Sequence (5'→3')** |
| --- | --- |
| MiIDA1-F | GGTCCTTCACGGAAACACAATG |
| MiIDA1-R | GGGAAGAAGAAGAAGGGGAAAA |
| MiIDA2-F | GGTCTCCTATTCCGCCTTCTG |
| MiIDA2-R | CCAATTGAGAGAGAACCGCCTA |
| MiPG-F | CACGTGGAACTTCTGCTAGAGT |
| MiPG-R | AGATCCTCCCTGCCATGTTTTT |
| MiPL-F | GGTTGACTCCATGACTTCTGGT |
| MiPL-R | TTGTGGGATGACTGGTGATACG |
| MiEXP-F | GTCCAGGTCGACTACTAAGAGC |
| MiEXP-R | ACAATGATTACATTCGTTCACG |
| MiCaSy3-F | AGACTGTATCTGTTGGAAGGCG |
| MiCaSy3-R | CTTGCGCTGTCATATGTGGAAG |
| MiCeSy-F | TAAACCCATTTGTCAACCGGGA |
| MiCeSy-R | TCTCTTCAGCAACTCCCCATTT |
| MiCOBRA4-F | TGGTACTCTTTGTAGCTTCTGCA |
| MiCOBRA4-R | TTAAACCAAGCGGCACATCAAG |
| MiETR1-F | TGAGGAGCGTTTTATTAGGCCT |
| MiETR1-R | TTTTTCCAGGCTCAATGGCATC |
| MiERS1-F | AGAAGCCTTTAGGTCACAGTGA |
| MiERS1-R | TCGGCCTTCAGCTTTTACTTGA |
| MiETR2-F | CTACTGCAAGGAATCGCCAATG |
| MiETR2-R | GGAGACAAAATGGTGTTGTCGG |
| MiEIN3-F | ATGGATAGCCTACCAAAGCAGG |
| MiEIN3-R | TTCAACAATGTCTGCTCCTCGA |
| MiCTR1-F | AAGGCCCTTGATCAAACCTCTT |
| MiCTR1-R | AACTCTTCGGCGTTACTCAAGT |
| MiERF113-F | CTTCATCGGGTTCTGATCCTAGA |
| MiERF113-R | CCGTTACTAAAACAAACCTTCTCCA |
| MiERF3-F | AAGCCTCTTGAAGATGACCACA |
| MiERF3-R | AACTGAGATTTTGTTGGCCTGC |
| MiGAPDH-F | GTGACCAGAGGCTACTTGATGCTAG |
| MiGAPDH-R | GCTGCACCAGTCGAAGTTGGAAC |
